# Direct terrestrial test of Lorentz symmetry in electrodynamics to 10^−18^

**DOI:** 10.1038/ncomms9174

**Published:** 2015-09-01

**Authors:** Moritz Nagel, Stephen R. Parker, Evgeny V. Kovalchuk, Paul L. Stanwix, John G. Hartnett, Eugene N. Ivanov, Achim Peters, Michael E. Tobar

**Affiliations:** 1 Institut für Physik, Humboldt-Universität zu Berlin, Newtonstrasse 15, 12489 Berlin, Germany; 2 School of Physics, The University of Western Australia, 35 Stirling Highway, Crawley, Western Australia 6009, Australia; 3 Institute for Photonics and Advanced Sensing, School of Physical Sciences, University of Adelaide, Adelaide, South Australia 5005, Australia

## Abstract

Lorentz symmetry is a foundational property of modern physics, underlying the standard model of particles and general relativity. It is anticipated that these two theories are low-energy approximations of a single theory that is unified and consistent at the Planck scale. Many unifying proposals allow Lorentz symmetry to be broken, with observable effects appearing at Planck-suppressed levels; thus, precision tests of Lorentz invariance are needed to assess and guide theoretical efforts. Here we use ultrastable oscillator frequency sources to perform a modern Michelson–Morley experiment and make the most precise direct terrestrial test to date of Lorentz symmetry for the photon, constraining Lorentz violating orientation-dependent relative frequency changes Δ*ν*/*ν* to 9.2±10.7 × 10^−19^ (95% confidence interval). This order of magnitude improvement over previous Michelson–Morley experiments allows us to set comprehensive simultaneous bounds on nine boost and rotation anisotropies of the speed of light, finding no significant violations of Lorentz symmetry.

## Abstract (web summary)

Lorentz invariance, tested more than a century ago by Michelson and Morley, is a foundational property of modern physics within the standard model and general relativity. Here, the authors report the most precise terrestrial test to date, bounding the lack of violations of Lorentz symmetry in photons to 10^–18^.

## Graphical abstract

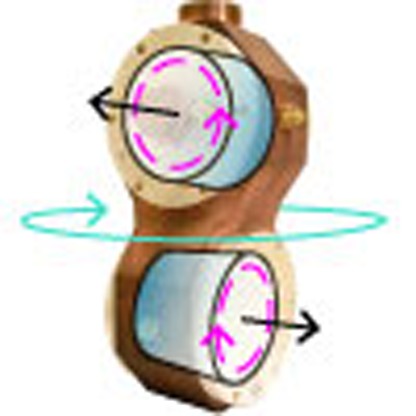

A significant consequence of Lorentz symmetry is the isotropic nature of the speed of light, which remains invariant under rotation and boost transformations. Measuring the isotropy of the speed of light has played an important role in physics, starting with the seminal Michelson and Morley interferometer experiment in the late nineteenth century^1^. What began as a hunt for a luminiferous ether soon evolved into tests of the revolutionary theory of special relativity. Current motivation arises from the search for hints of new physics^2^,^3^,^4^, to provide direction in the quest for a unified theory of quantum mechanics and general relativity^5^.

Despite the success of unifying the weak force and electrodynamics^6^, unification of the standard model with gravity remains elusive. Many approaches invoking string theory^7^, quantum gravity^8^,^9^,^10^ and noncommutative field theories^11^ either explicitly require or naturally permit Lorentz symmetry to be broken^5^. Efforts to test these theories experimentally focus on searches for violations of Lorentz and charge–parity–time (CPT) symmetry, which are expected to occur at the Planck scale (5.4 × 10^−44^ s, 1.6 × 10^−35^ m and 1.2 × 10^19^ GeV), with suppressed effects manifesting in regimes experimentally accessible via precision measurement^12^. Many tests of Lorentz and CPT symmetry have been performed for a variety of fields, particles and forces, with no violations reported to date^13^.

Just as the theories motivating Michelson–Morley style experiments have changed, so too has the technology used. The transition from conventional optical interferometer searches to resonator-stabilized frequency source tests^14^,^15^,^16^,^17^,^18^,^19^ has enabled a rapid increase in experimental sensitivity; an overview is provided in Fig. 1. The exceptional frequency stability performance of these modern sources makes tests of Lorentz symmetry of the photon one of the most powerful experimental tools in the search for clues towards unification frameworks such as quantum gravity.

Here we present the results of the most sensitive Michelson–Morley style frequency comparison experiment performed to date. We use 1 year of data to set new bounds on the nine possible rotational and boost isotropies of the speed of light, with our results expressed as constraints on coefficients of the standard model extension (SME)^20^. We find no evidence of any statistically significant violation of Lorentz symmetry of the photon.

## Results

### Experiment design

**Figure 2:**
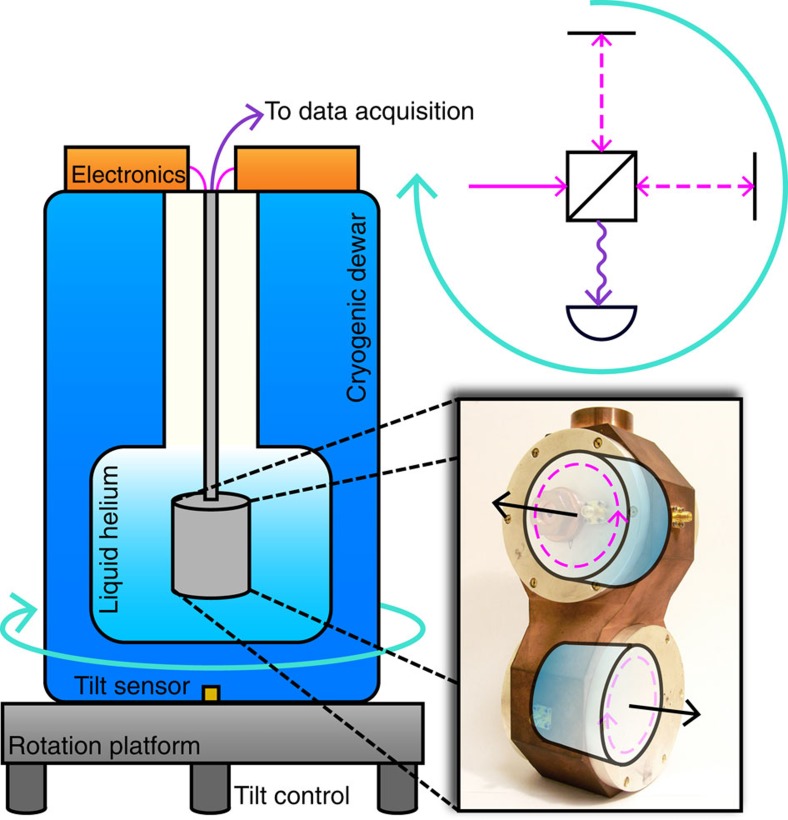
Schematical overview of the experimental setup. Two sapphire cylinders were loaded in a dual-cavity mount with their crystal axes (black arrows) aligned orthogonally. This was housed within two vacuum cans and cooled to 4 K in a liquid helium dewar. A whispering gallery mode resonance was excited in each sapphire, the Poynting vector is shown by the dashed magenta arrow. Microwave and DC electronics were used to create two loop oscillators, each locked to the resonance of a sapphire. The two oscillators were beat against each other and the difference frequency was recorded. The apparatus was continuously rotated with a 100 s period on a tilt-controlled air-bearing turntable. Comparison with the original Michelson–Morley arrangement is presented in the top right to demonstrate experimental concept.

**Figure 3:**
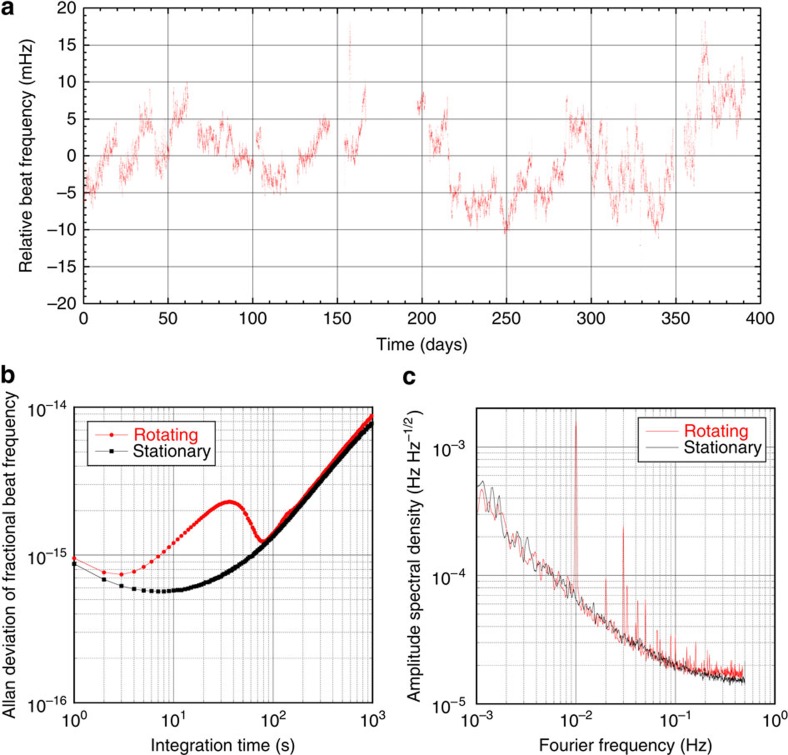
Beat frequency of the two cryogenic oscillators. (**a**) Sampled beat frequency recorded over the course of the experiment from June 2012 to June 2013, with linear drift (∼86 μHz per day) and a 347 kHz offset subtracted for display purposes. The gap in data was because of a short period of downtime during the 2012/2013 transition. (**b**) Allan deviation of the fractional beat frequency, demonstrating the fundamental frequency stability of the oscillators when they are stationary in the laboratory (black trace) compared with the stability when actively rotated (red trace). Rotated stability represents typical performance and was calculated from a subset of the data taken during April 2012. (**c**) Comparison of amplitude spectral density of the oscillators beat frequency when stationary (black trace) and rotated (red trace) in the laboratory, computed from the same data as in **b**. Peaks corresponding to the fundamental turntable rotation frequency and higher-order harmonics can be resolved clearly, whereas the background noise level has not increased. Signals of interest are located at sidereal and annual sideband frequencies around the second turntable harmonic (2*ω*_R_=0.02 Hz), where the noise is not increased by rotation.

A schematic of our Michelson–Morley oscillator experiment, located in Berlin (latitude 52.4°), is presented in Fig. 2. Two cylindrical copper cavities were each loaded with a nominally identical cylindrical sapphire dielectric crystal. Whispering gallery modes were excited within the crystals with a resonance frequency of 12.97 GHz. Pound control electronics are used to build two loop oscillator circuits, with each oscillator locked to the resonance frequency of a cavity^21^. The cavity crystal axes were aligned perpendicular to each other such that the Poynting vectors and thus path of light propagation for the resonant modes were orthogonal to each other (see insert of Fig. 2). A fractional change in the speed of light would induce a proportional fractional change in the beat note frequency of the two oscillators. Thus, to be highly sensitive to the signals of Lorentz invariance violation (LIV) one needs to employ extremely low-noise frequency sources. Of course, systematic noise sources also lead to frequency changes; thus, we require both low-noise sources and a well-controlled setup. At cryogenic temperatures, the frequency stability of the loop oscillator circuit was optimized, which ultimately dictated the sensitivity of the experiment (see Fig. 3).

We analysed the experiment relative to a sun-centred inertial reference frame^22^. As such, passive rotation transformations were provided courtesy of Earth's daily and annual cycles. In addition, the apparatus was actively rotated in the laboratory on a tilt-stabilized high-precision air-bearing turntable that was run with a period of 100 s. This corresponds to the optimal performance of the experiment, which is a trade-off between oscillator frequency stability, rate of data acquisition and the influence of systematic noise. A 200 l liquid helium reservoir allowed for 3 weeks of continuous operation before refilling was required.

Owing to the symmetry of the setup with respect to the sun-centered inertial reference frame, the signal of interest occurs at twice the turntable rotation frequency, 2*ω*_R_, with additional sideband modulations arising from Earth's sidereal rotation, *ω*_⊕_, and orbit, Ω_⊕_. This has the added benefit of suppressing the influence of any rotation-induced sources of systematic noise that manifest at the fundamental turntable rotation frequency (see Fig. 3). The experimental setup is first-order sensitive to LIVs of rotational transformations, with a suppressed sensitivity to symmetry breaking of boost transformations. The suppression is of order 10^−4^, which is the ratio of Earth's orbital velocity to the speed of light.

### Analysis

Data were collected over the course of a year; the beat frequency between the oscillators for the duration of the experiment is displayed in Fig. 3. The beat frequency data were analysed for periodic signals of variation corresponding to modulation frequencies of interest. Least-squares regression was used to perform a fit to the fundamental turntable rotation frequency and the first harmonic,


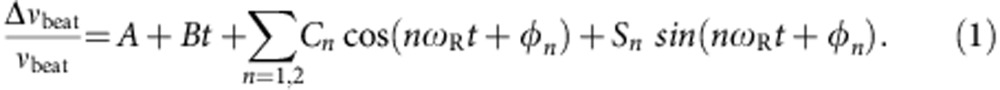


Error-weighted least-squares regression was then used to fit the amplitudes *C*_*n*_ and *S*_*n*_ from equation (1) to the daily variations (*ω*_⊕_, 2*ω*_⊕_) and finally to the annual frequencies (Ω_⊕_, 2Ω_⊕_). A detailed description of the data analysis process can be found in previous work^19^,^23^,^24^ and in the Methods section.

Taking error-weighted averages of relevant amplitudes from equation (1) we found a 2*ω*_R_ amplitude of −98±6 nHz. This value of interest, 2*ω*_R_, is only statistically significant, owing to the influence of systematic noise sources (see Fig. 3), the most dominant of which is the dependency of oscillator resonance frequency on external magnetic fields, arising from the presence of impurities in the sapphire crystal^25^ and ferrite-based microwave components. The frequency variations induced by moving the oscillators through the quasi-static magnetic field of the Earth in the laboratory are indistinguishable from a Lorentz violating signal. However, the leakage into sidereal and annual sidebands is negligible.

Calculating the weighted average of quadrature amplitudes for daily and twice daily variations (*ω*_⊕_, 2*ω*_⊕_; see Supplementary Figs 1–8), we found a frequency variation of 12±14 nHz (95% confidence interval), leading to our reported bound on the overall sensitivity of the experiment, Δ*ν*/*ν*≤9.2±10.7 × 10^−19^ (95% confidence interval). The presence of a statistically significant LIV signal in any solitary amplitude would propagate through to the final value.

## Discussion

We use our data to place limits on coefficients of the SME^20^, which is an effective field theory framework containing the standard model and general relativity along with any possible Lorentz and CPT symmetry violating coefficients that could arise from the various fields. It is important to note that the SME is a purely phenomenological framework, designed to enable comprehensive searches for Lorentz and CPT symmetry violations and facilitate cataloguing and comparisons between experiments. It is conventional to express results in a single inertial reference frame; in this case, the widely adopted sun-centred celestial equatorial frame is used^22^.

It is important to point out that technically, at a fundamental level, a change in the beat frequency of the experiment is proportional to not just a change in the speed of light (owing to Lorentz violation of the photon) but also to changes in the propagating medium, owing to Lorentz violation of the electron^26^,^27^,^28^. What we are actually measuring and constraining is a difference or combination of these potential effects. Owing to the choice of coordinates, the proton sector remains Lorentz invariant and does not contribute to any changes in the propagating medium^22^.

We restrict our attention to the photon sector of the minimal SME^22^, which only contains operators of renormalizable dimension in flat spacetime. The resulting possible violations of Lorentz symmetry can be divided into polarization-dependent or -independent effects; astrophysical constraints^29^ have limited polarization-dependent violations far beyond the reach of this work and are thus ignored. What remains are nine coefficients constraining different effects—rotation violations, described by the five coefficients of the 3 × 3 symmetric traceless matrix 
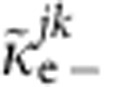
, boost violations, described by the three coefficients of the 3 × 3 antisymmetric matrix 
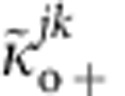
 and an overall isotropic deviation of *c*, described by the scalar 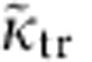 (ref. 24). Experimental sensitivities to these coefficients are provided in Supplementary Table 2.

For sapphire with the *c* axis parallel to the crystal axis, as is the case in this work, the sensitivity to Lorentz violation of electrons is reduced^27^,^28^. The effect is such that the orientation coefficients, 
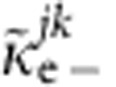
, and the isotropic deviation, 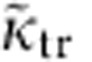, should be replaced with 
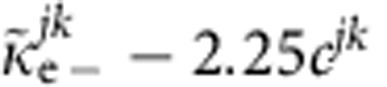
 and 
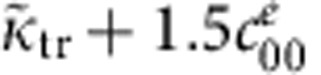
, respectively. In presenting the results of this work, we adhere to established convention and assume that the electron sector remains Lorentz invariant, as it has been for all modern cavity-based Michelson–Morley experiments.

Bounds for coefficients of the minimal SME are presented in Table 1. It is important to note that 
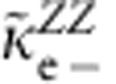
 is constrained solely by the amplitude of the cosine variation at twice the turntable frequency, 2*ω*_R_, which is dominated by known systematic noise processes (magnetic field). Therefore, the presented bound is obtained by taking the statistical mean of the time-dependent *C*_2_ amplitudes from equation (1), with the error given by the s.d. of the amplitudes. All coefficients are statistically insignificant; we report no evidence for violations of Lorentz symmetry in electrodynamics.

For the rotation symmetry breaking coefficients, 
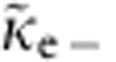
, our bounds are comparable to recent results from a trapped ion experiment^30^ that put constraints on four sets of linear combinations of electron and photon coefficients. In contrast, our reported bounds on the five 
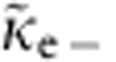
 coefficients were set under the assumption that Lorentz invariance of the electron is conserved (that is, *c*^*jk*^=0); ultimately, this works derives bounds from nine linear combinations of photon and electron coefficients (see Supplementary Table 2), which in principle offers better opportunities for disentangling coefficients. Compared with bounds derived from previous modern Michelson–Morley cavity tests^18^,^19^, where it was also assumed that the electron remained Lorentz invariant, we improve by up to a factor of ∼4.

The trapped ion experiment^30^ was not sensitive to the isotropic deviation, 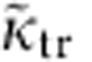, or the boost symmetry breaking coefficients, 
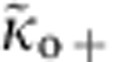
. For 
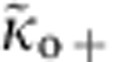
, we improve on the current state-of-the-art^13^,^18^,^19^ by up to a factor of ∼5. We also improve on our existing bounds^24^ for the isotropic shift, 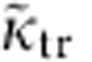, by a factor of ∼20, as recent work^31^ demonstrated that double-pass asymmetric resonators constructed out of a single material are only sensitive to higher-order coefficients (*d*>4) of the SME.

Despite thoughtful advances in the implementation and design of alternative experiments, cavity-based Michelson–Morley tests such as the one presented in this work still remain the most powerful and comprehensive way to search for non-birefringent LIV effects in electrodynamics.

Scheduled upgrades to the experiment to improve the fundamental stability of the microwave oscillators^32^ will soon allow for even more sensitive tests. The installation of superior magnetic shielding around the oscillators will reduce the influence of systematic noise sources. The addition of a separate optical cavity system in the same cryogenic environment will also open up opportunities for exploring additional higher-order coefficients^31^,^33^ and matter sector coefficients^34^, allowing a more complete disentangling of electron and photon sector coefficients.

A modern Michelson–Morley experiment has been performed with two orthogonally aligned stable microwave oscillators. Using data of the beat note frequency between the two oscillators recorded over the course of a year, we are able to constrain LIV-induced Δ*ν*/*ν* to be <10^−18^, the most precise measurement ever made for electromagnetic cavity experiments. No violations of Lorentz symmetry were observed.

## Methods

### Experiment

Two nominally identical cylinders (51 mm diameter and 30 mm height) of HEMEX grade ultra-pure sapphire (GT Advanced Technologies) were machined from the same boule. The crystals were cleaned in a solution of 70 % nitric acid containing several drops of hydrofluoric acid and then mounted in copper cavities and sealed in a stainless steel vacuum can, evacuated to sub-10^−6^ mbar. Whispering gallery resonant mode WGE_16,0,0_ was used for both cavities. This mode features a dominant radial electric field with 32 variations around the circumference of the sapphire crystal. The majority of the electromagnetic fields are contained within the sapphire dielectric, with minimal evanescent field leaking out to the copper walls of the cavity structure. The first cavity had a resonant frequency of 12.9688 GHz and a loaded quality factor of 10^9^ at 4.4 K, while the second cavity had a resonant frequency of 12.9685 GHz and a loaded quality factor of 1.5 × 10^9^ at 4.4 K.

Small concentrations of impurities within the sapphire give rise to a temperature/frequency turning point^21^; for the 347 kHz beat frequency between the two resonators, this turning point occurred at 5.5 K. A temperature controller (model 340, Lake Shore Cryotronics, Inc.) was used in conjunction with a resistive heater and a carbon-glass temperature sensor to operate the resonators at the turning point. Custom microwave circuits and control electronics are used to create a loop oscillator out of each resonator. Pound locking is employed, whereby modulation sidebands reflected back from the resonator are demodulated with a lock-in amplifier (model SR830, Standford Research Systems) and from this a correction signal is applied to a voltage-controlled phase shifter to align the frequency of the oscillator with that of the resonator. Power incident on the cavity is monitored with a detector (model DT8016, Herotek, Inc.), the signal is compared against a user-defined set-point and a correction voltage is applied to a voltage-controlled attenuator placed *in situ* with the loop oscillator. The oscillator comparison beat frequency was logged on a frequency counter (model 53142A, Agilent Technologies, Inc.) referenced to a 10 MHz rubidium standard.

The oscillators were rotated on a high-precision air-bearing rotation table (Kugler GmbH); an 18,000 point optical encoder was used to track the angular position of the table and maintain a constant rotation velocity. The table sat on three aluminium legs, with each one in turn placed on a force sensor; these were used to align the centre of mass with the axis of rotation. A biaxial high-gain tilt sensor (model 755, Applied Geomechanics) sits at the centre of the experiment. Variations in tilt were compensated for by heating or cooling two of the three aluminium legs. All three legs were heated above ambient temperature to improve performance of the tilt-control system.

### Data analysis

Original time tags are converted into time in seconds since the Vernal Equinox before the start of data collection (20 March 2012, 05:14 UTC+0). This format assists with calculating the relevant phase offsets required to analyse the data in the context of the SME^22^. The rotation turntable features an optical encoder with 18,000 points and a trigger mark to indicate that a full rotation has occurred; the data are scanned and incomplete rotations are discarded. The rotation points are converted into a modular angle value in radians. The data are broken up into subsets containing ten full turntable rotations (corresponding to ∼1,000 s) and an ordinary least-squares regression is used to fit the subset to the following model:


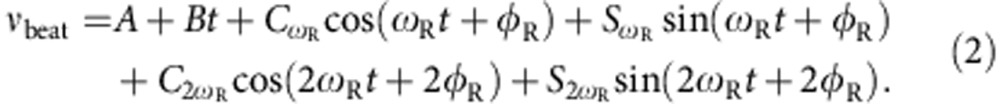


The value of *ω*_R_*t* comes straight from the modular angular position of the turntable recorded in the data. The phase offset, *φ*_R_, is the angular difference between the start of data collection and the alignment of the crystal axis of the top cavity with geographical East. The start of data collection is triggered at the same point for each experimental run. The phase offset is once again only needed to aid with the reporting of coefficients in the SME framework. The amplitudes of equation (2) and the mean time of each subset are stored to file, producing 19,597 entries. A histogram of the magnitude of errors for the fits to 
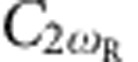
 and 
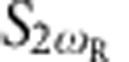
 is produced (Supplementary Fig. 9); all subsets with an error further than 3*σ* from the mean are discarded, resulting in 299 entries being removed (1.5% of the full data set). These points correspond to data with excessive additional noise present that does not fit our model or expected signals and would otherwise corrupt the quality of the subsequent analysis.

The demodulated data set is then broken up into subsets containing 100 entries each (∼1.2 days) and fit to the following model via a weighted least-squares regression, using the square of the s.e. of the fits from equation (2) as the weights.


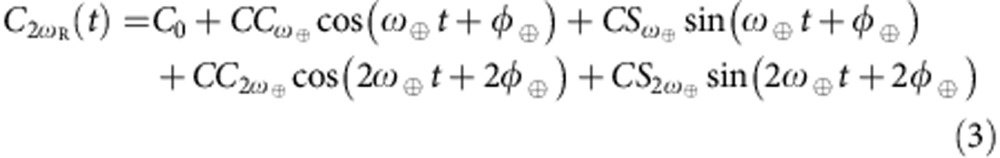



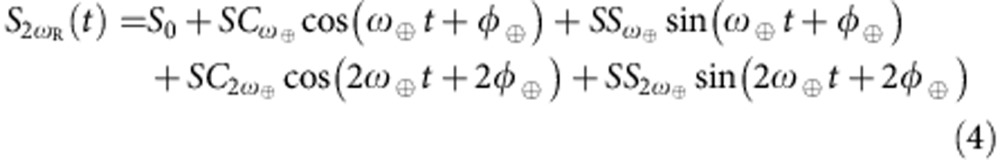


The phase offset *φ*_⊕_ is the difference between the 2012 Vernal Equinox and the alignment of geographical East with the *Y* axis of the Sun Centred frame used for determination of the SME coefficients^22^. The value of *ω*_⊕_ used was 7.3 × 10^−5^ rad s^−1^ and *t* is the relevant mean time calculated during the previous demodulation (equation (2)). Once again the amplitudes, s.e. and mean time for each subset is stored to file. The fitted amplitudes and s.e. from equations (3) and (4) are shown in Supplementary Figs 1–8. The eight amplitudes from equations (3) and (4) representing the first two harmonics of daily variations are used to bound the overall sensitivity of the experiment to LIVs. We take the s.e.-weighted average of all the amplitudes, which is equivalent to weighting by the variance. Noting the distribution of the histogram of all the amplitudes (Supplementary Fig. 10), whereby 95% of the points lie within 2 s.d. of the mean, we multiply the associated s.e. by 2, to determine the 95% confidence interval for our bound, Δ*ν*/*ν*≤9.2±10.7 × 10^−19^.

The final stage of the data analysis is used to set bounds on coefficients of the SME. Each amplitude and s.e. from equations (3) and (4) is used to perform a weighted least-squares regression fit to an offset and variations at harmonics of Earth's orbital frequency Ω_⊕_ and 2Ω_⊕_. Supplementary Table 2 summarizes the relevant amplitudes, their sensitivity to different coefficients of the SME and the corresponding numerical weights.

For SME coefficients where more than one bound is derived from Supplementary Table 2, we report the error-weighted average of all contributions. The coefficient 
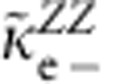
 is only accessible via the second harmonic of turntable rotation (amplitude 
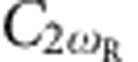
 in equation (2), or offset *C*_0_ in equation (3)), where systematic noise sources are present. The bound for this coefficient is set by taking the statistical mean and s.d. of all the values obtained for *C*_0_, noting that the bound should be consistent with a null result for systematic noise with a random phase, while a substantial Lorentz violating signal with constant phase would be statistically significant.

## Additional information

**How to cite this article:** Nagel, M. *et al*. Direct terrestrial test of Lorentz symmetry in electrodynamics to 10^−18^. *Nat. Commun.* 6:8174 doi: 10.1038/ncomms9174 (2015).

## Supplementary Material

Supplementary Information Supplementary Figures 1-10, Supplementary Tables 1-2 and Supplementary References

## Figures and Tables

**Figure 1:**
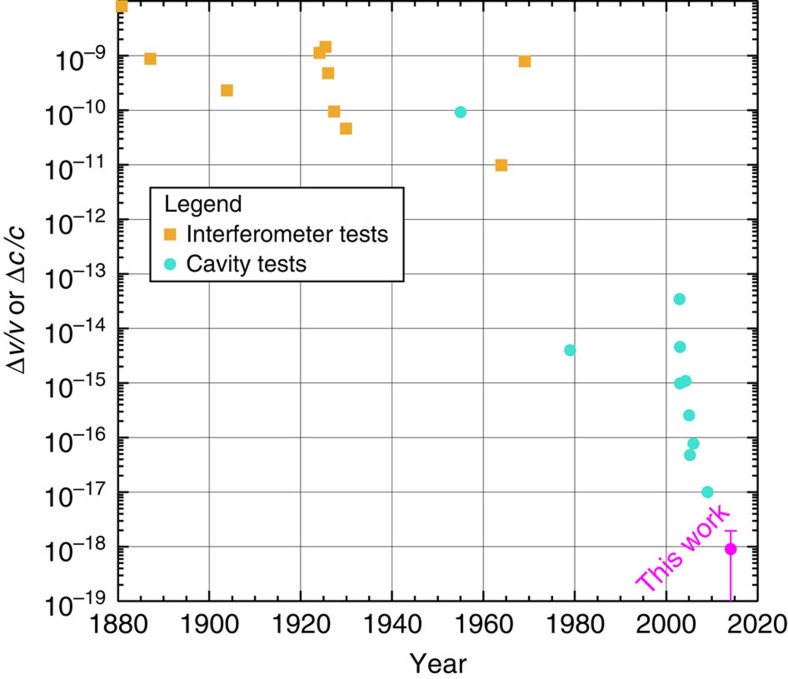
Historical overview of Michelson–Morley style tests of Lorentz invariance in electrodynamics. Presented are interferometer tests (squares) that measure fractional shifts in the speed of light, Δ*c*/*c*, and cavity-based tests (circles) such as this work that measure a fractional change in frequency, Δ*ν*/*ν*. Bounds are taken directly from the results reported in original publications. A full list is provided in the Supplementary References, with numerical values given in Supplementary Table 1.

**Table 1:** Bounds on non-birefringent photon-sector coefficients of the minimal SME.

Coefficient	Bound (Error)
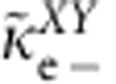	−0.7 (1.6)
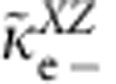	−5.5 (4.0)
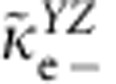	−1.9 (3.2)
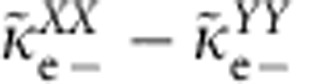	−1.5 (3.4)
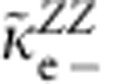	−286 (279)
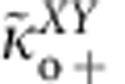	−3.0 (3.4)
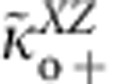	0.2 (1.7)
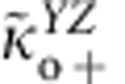	−2.0 (1.6)
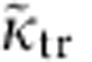	−6.0 (4.0)

SME, standard model extension.

Errors are standard 1*σ* of statistical origin. Values for 
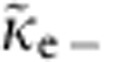
 are given in 
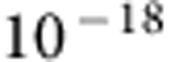
, 
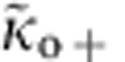
 in 10^−14^ and 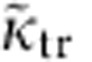 in 10^−10^.
