# Supplementary material for: Direct terrestrial test of Lorentz symmetry in electrodynamics to 10−18
Source: Nat Commun. 2015 Sep 1;6:8174. doi: 10.1038/ncomms9174 (PMC4569797; doi:10.1038/ncomms9174)
Supplement: Supplementary Information — Supplementary Figures 1-10, Supplementary Tables 1-2 and Supplementary References [file ncomms9174-s1.pdf]

Supplementary Figures

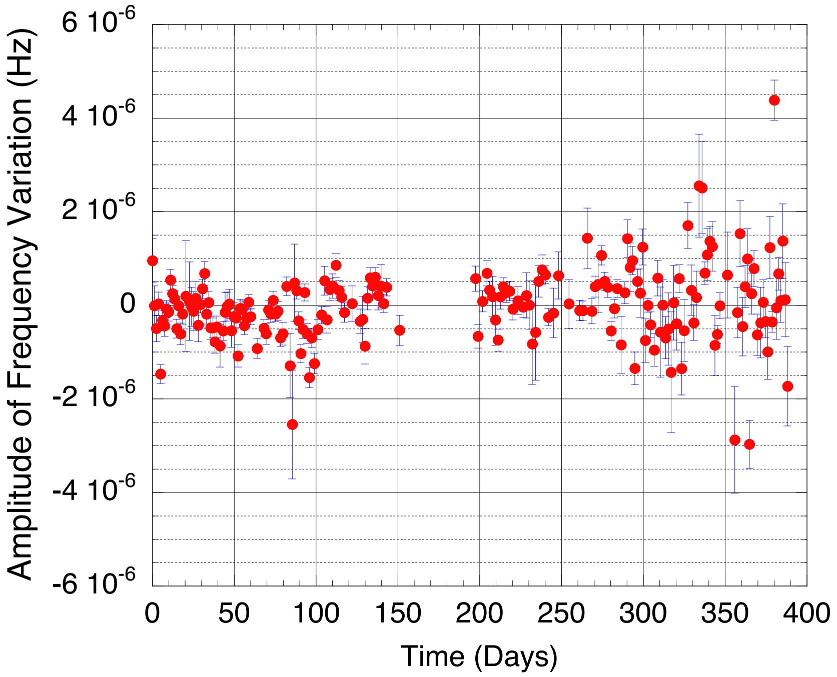

Supplementary Figure 1: **Subset fits to sidereal amplitude** $CC_{\omega_\oplus}$ from equation (3) obtained as discussed in the main text. Statistical 1σ error bars are shown in blue.

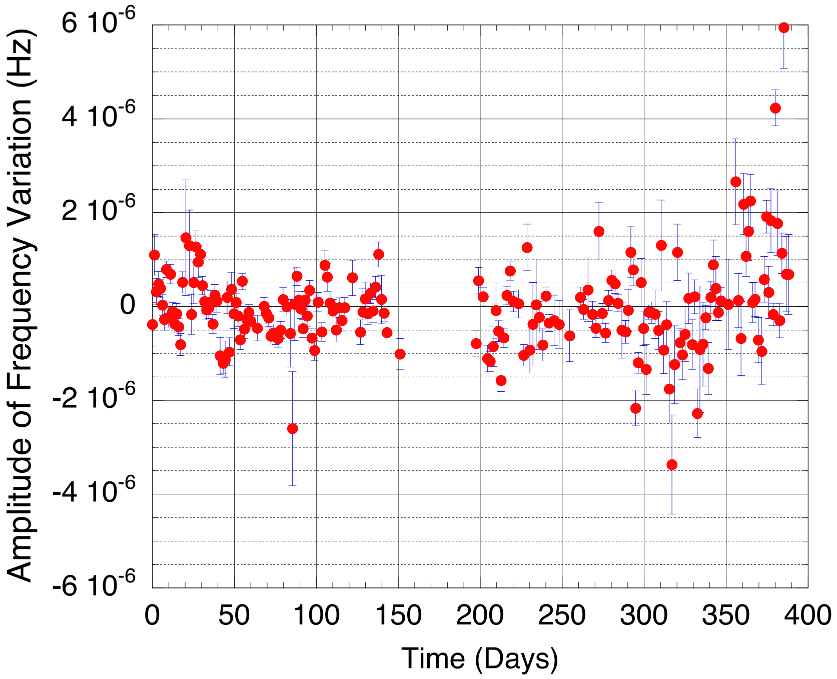

Supplementary Figure 2: **Subset fits to sidereal amplitude** $CS_{\omega_\oplus}$ from equation (3) obtained as discussed in the main text. Statistical 1σ error bars are shown in blue.

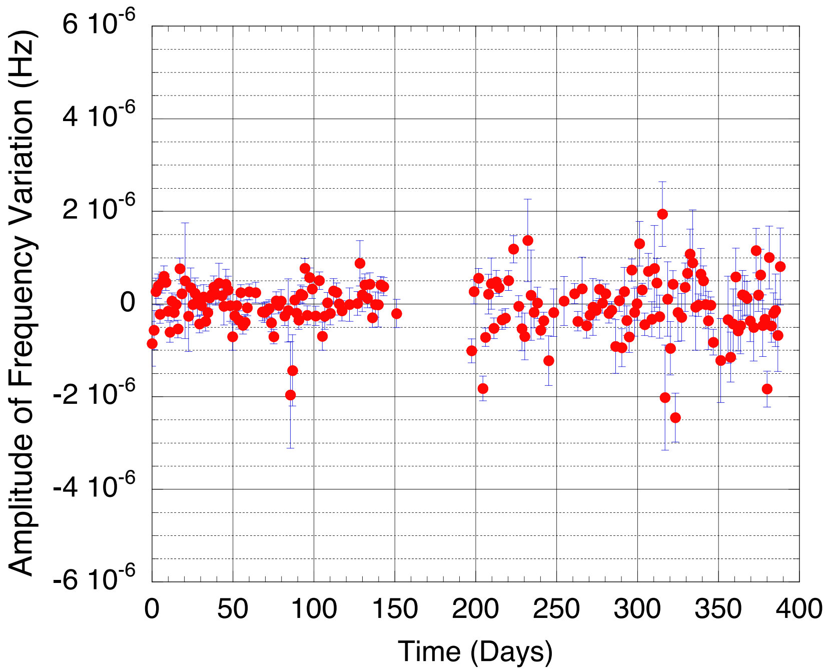

Supplementary Figure 3: **Subset fits to sidereal amplitude** $CC_{2\omega_\oplus}$ from equation (3) obtained as discussed in the main text. Statistical 1σ error bars are shown in blue.

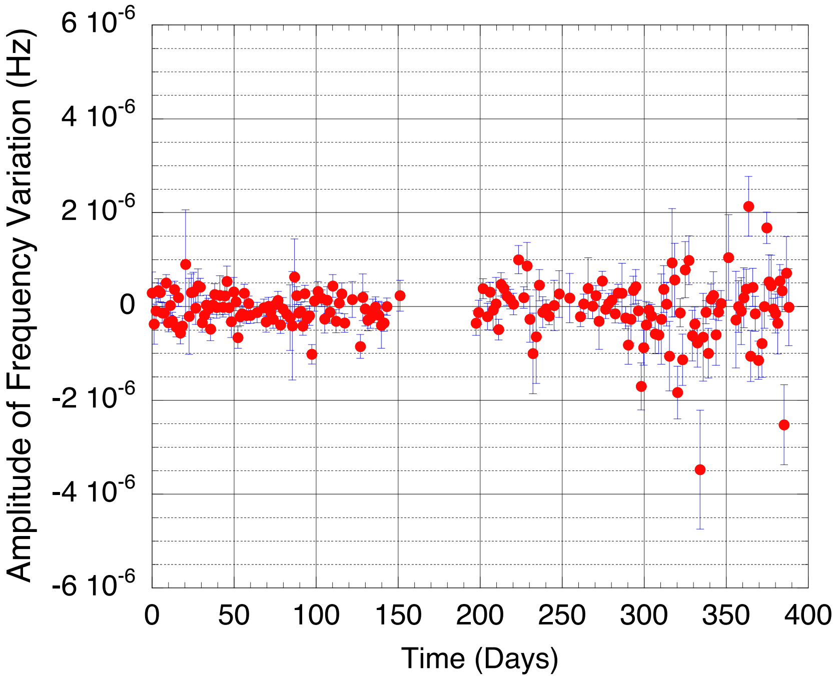

Supplementary Figure 4: **Subset fits to sidereal amplitude** $CS_{2\omega_\oplus}$ from equation (3) obtained as discussed in the main text. Statistical 1σ error bars are shown in blue.

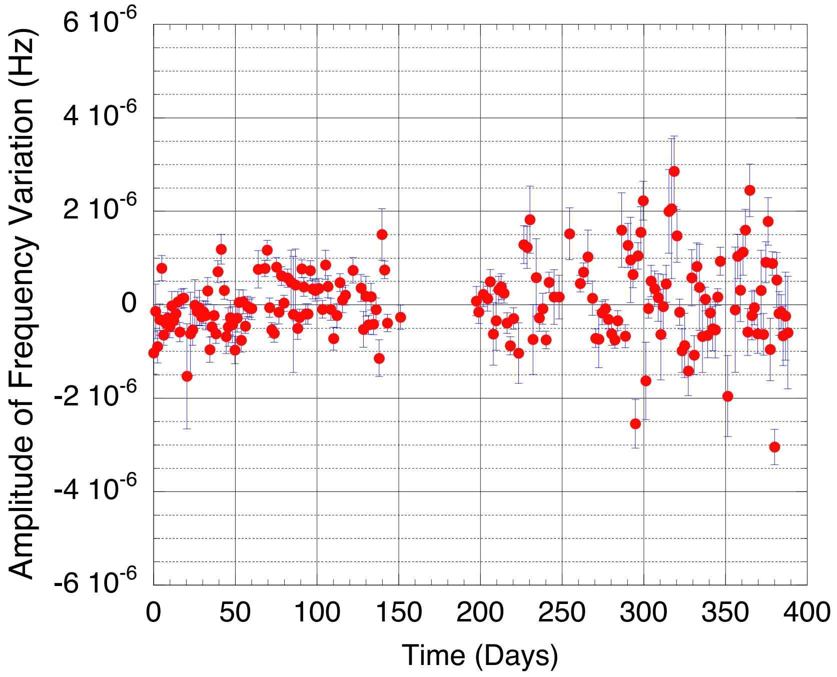

Supplementary Figure 5: **Subset fits to sidereal amplitude** $SC_{\omega_\oplus}$ from equation (4) obtained as discussed in the main text. Statistical 1σ error bars are shown in blue.

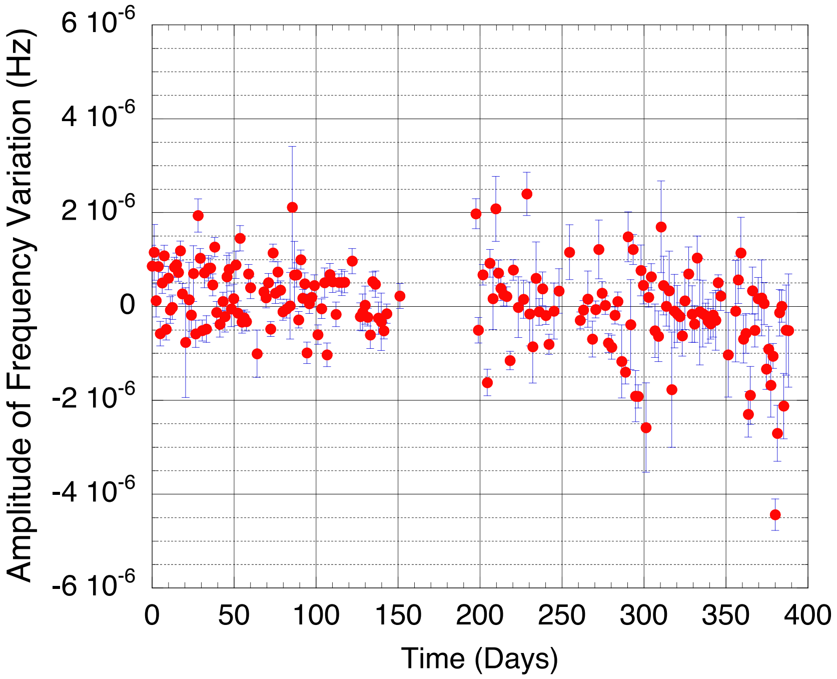

Supplementary Figure 6: **Subset fits to sidereal amplitude** $SS_{\omega_\oplus}$ from equation (4) obtained as discussed in the main text. Statistical 1σ error bars are shown in blue.

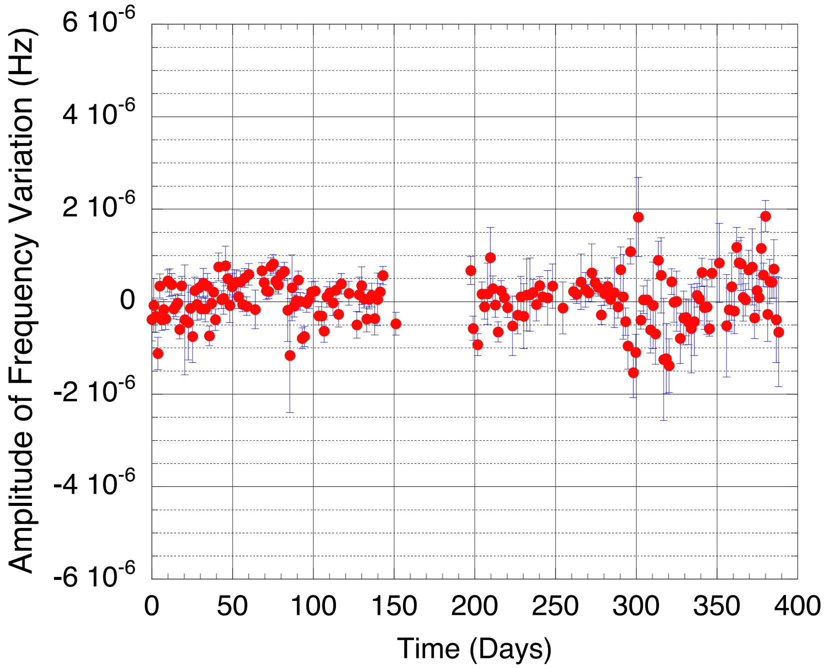

Supplementary Figure 7: **Subset fits to sidereal amplitude** $SC_{2\omega_\oplus}$ from equation (4) obtained as discussed in the main text. Statistical 1σ error bars are shown in blue.

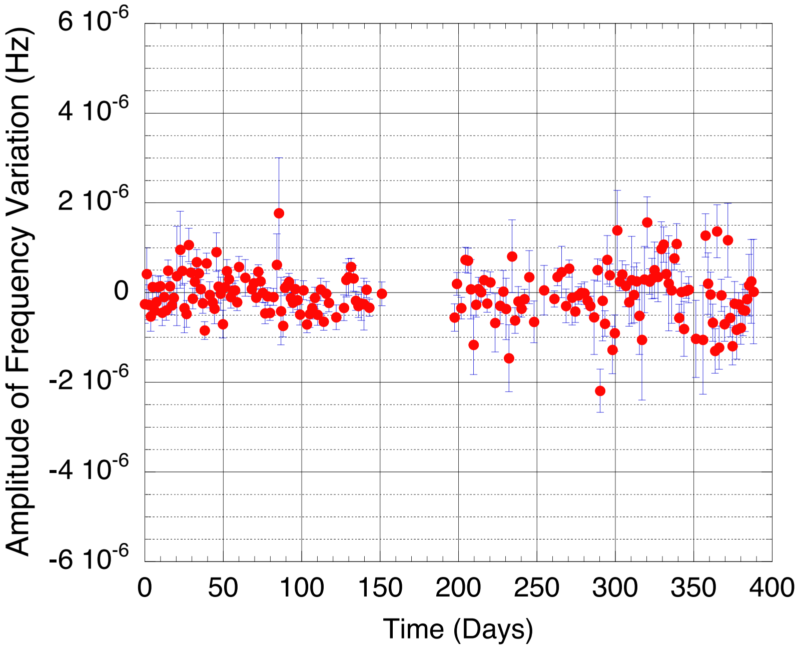

Supplementary Figure 8: **Subset fits to sidereal amplitude** $SS_{2\omega_\oplus}$ from equation (4) obtained as discussed in the main text. Statistical 1σ error bars are shown in blue.

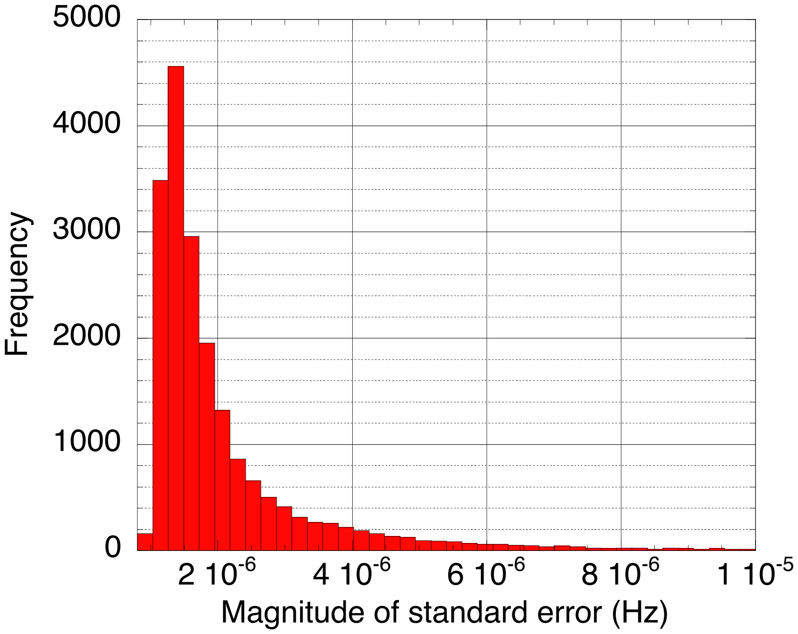

Supplementary Figure 9: **Statistical histogram of the magnitude of the standard errors** for fits to the amplitudes of the $2\omega_R$ components of equation (2) in the main text. Values are from fits to subsets of data ~1000 seconds (10 rotations) long.

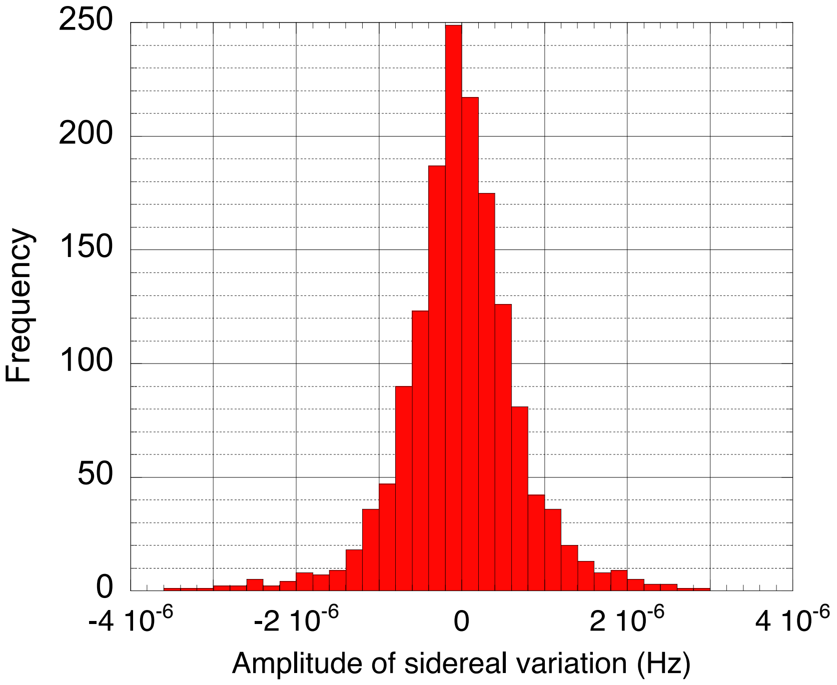

Supplementary Figure 10: **Statistical histogram of amplitudes for $\omega_\oplus$ and $2\omega_\oplus$ variations**, given by the 8 time-varying components of equations (3) and (4) in the main text. Values are from fits to subsets of data $\sim$1.2 days long.

Supplementary Tables

Supplementary Table 1: **Historical overview of Michelson-Morley style experiments**, constraining Lorentz invariance in electrodynamics as presented in Figure (1) of the main text.

Year	Type	$\Delta c/c$ or $\Delta\nu/\nu$	Reference
1881	Interferometer	8.3E-9	[1]
1887	Interferometer	9.1E-10	[2]
1904	Interferometer	2.3E-10	[3]
1924	Interferometer	1.2E-9	[4]
1925	Interferometer	1.4E-9	[5]
1926	Interferometer	5E-10	[6]
1927	Interferometer	1E-10	[7]
1930	Interferometer	4.8E-11	[8]
1955	Cavities	1E-10	[9]
1964	Interferometer	1E-11	[10]
1969	Interferometer	8.1E-10	[11]
1979	Cavities	4E-15	[12]
2003	Cavities	3.4E-14	[13]
2003	Cavities	1E-15	[14]
2003	Cavities	4.3E-15	[15]
2004	Cavities	1.1E-15	[16]
2005	Cavities	2.6E-16	[17]
2005	Cavities	5E-17	[18]
2006	Cavities	8E-17	[19]
2009	Cavities	1E-17	[20]
2009	Cavities	1E-17	[21]
2014	Cavities	1E-18	This work

Supplementary Table 2: **Amplitudes of Cosine / Sine frequency components of interest and their sensitivities and numerical weights**, calculating using the following values: $F_{1,2}$ describe combinations of electric and magnetic filling factors in the cavities (∼0.46,0.5), χ is the co-latitude of the experiment from the north pole ($\sim 38°$), η is the angle between Earth's orbital and equatorial planes (∼23.4°) and $\beta_\oplus$, Earth's orbital velocity suppressed by the canonical value for the speed of light in vacuum (9.9E-5). These sensitivities arise from orientation and design of the experiment and the frame transformations required to express bounds in the sun-centred frame of choice. Frame transformations and determination of sensitivities is discussed at length in the literature.

Amplitude	Sensitivity	Numerical Weight
S_0	-	-
$SS^0_{\omega_\oplus}$	$4F_1\sin(\chi)\tilde{\kappa}^{YZ}_{\mathrm{e}-}$	1.1
$SS_{\omega_\oplus}C_{\Omega_\oplus}$	$-4F_2\beta_\oplus\sin(\chi)\left(\cos(\eta)\tilde{\kappa}^{XY}_{\mathrm{o}+}-\sin(\eta)\tilde{\kappa}^{XZ}_{\mathrm{o}+}\right)$	4.9E-5 $\tilde{\kappa}^{XZ}_{\mathrm{o}+}$-1.1E-4 $\tilde{\kappa}^{XY}_{\mathrm{o}+}$
$SS_{\omega_\oplus}C_{2\Omega_\oplus}$	$-F_1\beta^2_\oplus\sin(2\eta)\sin(\chi)\tilde{\kappa}_{\mathrm{tr}}$	-2E-9
$SC^0_{\omega_\oplus}$	$-4F_1\sin(\chi)\tilde{\kappa}^{XZ}_{\mathrm{e}-}$	-1.1
$SC_{\omega_\oplus}S_{\Omega_\oplus}$	$4F_2\beta_\oplus\sin(\chi)\tilde{\kappa}^{XY}_{\mathrm{o}+}$	1.2E-4
$SC_{\omega_\oplus}C_{\Omega_\oplus}$	$4F_2\beta_\oplus\sin(\eta)\sin(\chi)\tilde{\kappa}^{YZ}_{\mathrm{o}+}$	4.8E-5
$SC_{\omega_\oplus}S_{2\Omega_\oplus}$	$2F_1\beta^2_\oplus\sin(\eta)\sin(\chi)\tilde{\kappa}_{\mathrm{tr}}$	2.2E-9
$SS^0_{2\omega_\oplus}$	$-4F_1\cos(\chi)\tilde{\kappa}^{XY}_{\mathrm{e}-}$	-1.5
$SS_{2\omega_\oplus}S_{\Omega_\oplus}$	$-4F_2\beta_\oplus\cos(\chi)\tilde{\kappa}^{XZ}_{\mathrm{o}+}$	-1.6E-4
$SS_{2\omega_\oplus}C_{\Omega_\oplus}$	$4F_2\beta_\oplus\cos(\eta)\cos(\chi)\tilde{\kappa}^{YZ}_{\mathrm{o}+}$	1.5E-4
$SS_{2\omega_\oplus}S_{2\Omega_\oplus}$	$2F_1\beta^2_\oplus\cos(\eta)\cos(\chi)\tilde{\kappa}_{\mathrm{tr}}$	6.6E-9
$SC^0_{2\omega_\oplus}$	$2F_1\cos(\chi)\left(\tilde{\kappa}^{XX}_{\mathrm{e}-}-\tilde{\kappa}^{YY}_{\mathrm{e}-}\right)$	7.2E-1
$SC_{2\omega_\oplus}C_{\Omega_\oplus}$	$-4F_2\beta_\oplus\cos(\eta)\cos(\chi)\tilde{\kappa}^{XZ}_{\mathrm{o}+}$	-1.5E-4
$SC_{2\omega_\oplus}S_{\Omega_\oplus}$	$-4F_2\beta_\oplus\cos(\chi)\tilde{\kappa}^{YZ}_{\mathrm{o}+}$	-1.6E-4
$SC_{2\omega_\oplus}C_{2\Omega_\oplus}$	$0.5F_1\beta^2_\oplus(3+\cos(2\eta))\cos(\chi)\tilde{\kappa}_{\mathrm{tr}}$	6.6E-9
C_0	$-3F_1\sin(\chi)^2\tilde{\kappa}^{ZZ}_{\mathrm{e}-}$	-5.1E-1
$CS^0_{\omega_\oplus}$	$4F_1\sin(\chi)\tilde{\kappa}^{YZ}_{\mathrm{e}-}$	8.8E-1
$CS_{\omega_\oplus}C_{\Omega_\oplus}$	$2F_2\beta_\oplus\sin(2\chi)\left(\sin(\eta)\tilde{\kappa}^{XZ}_{\mathrm{o}+}-\cos(\eta)\tilde{\kappa}^{XY}_{\mathrm{o}+}\right)$	3.9E-5 $\tilde{\kappa}^{XZ}_{\mathrm{o}+}$-8.9E-5 $\tilde{\kappa}^{XY}_{\mathrm{o}+}$
$CS_{\omega_\oplus}C_{2\Omega_\oplus}$	$2F_1\beta^2_\oplus\sin(\eta)\cos(\eta)\sin(\chi)\cos(\chi)\tilde{\kappa}_{\mathrm{tr}}$	1.6E-9
$CC^0_{\omega_\oplus}$	$4F_1\sin(\chi)\tilde{\kappa}^{XZ}_{\mathrm{e}-}$	8.8E-1
$CC_{\omega_\oplus}S_{\Omega_\oplus}$	$2F_2\beta_\oplus\sin(2\chi)\tilde{\kappa}^{XY}_{\mathrm{o}+}$	9.7E-5
$CC_{\omega_\oplus}C_{\Omega_\oplus}$	$-2F_2\beta_\oplus\sin(2\chi)\sin(\eta)\tilde{\kappa}^{YZ}_{\mathrm{o}+}$	-3.8E-5
$CC_{\omega_\oplus}S_{2\Omega_\oplus}$	$-F_1\beta^2_\oplus\sin(\eta)\sin(2\chi)\tilde{\kappa}_{\mathrm{tr}}$	-1.7E-9
$CS^0_{2\omega_\oplus}$	$-F_1(3+\cos(2\chi))\tilde{\kappa}^{XY}_{\mathrm{e}-}$	-1.5
$CS_{2\omega_\oplus}C_{\Omega_\oplus}$	$F_2\beta_\oplus(3+\cos(2\chi))\cos(\eta)\tilde{\kappa}^{YZ}_{\mathrm{o}+}$	1.5E-4
$CC^0_{2\omega_\oplus}$	$-0.5F_1(3+\cos(2\chi))\left(\tilde{\kappa}^{XX}_{\mathrm{e}-}-\tilde{\kappa}^{YY}_{\mathrm{e}-}\right)$	-7.4E-1
$CC_{2\omega_\oplus}C_{\Omega_\oplus}$	$F_2\beta_\oplus(3+\cos(2\chi))\cos(\eta)\tilde{\kappa}^{XZ}_{\mathrm{o}+}$	1.5E-4
$CC_{2\omega_\oplus}S_{\Omega_\oplus}$	$-F_2\beta_\oplus(3+\cos(2\chi))\tilde{\kappa}^{YZ}_{\mathrm{o}+}$	-1.6E-4
$CC_{2\omega_\oplus}C_{2\Omega_\oplus}$	$0.13F_1\beta^2_\oplus(3+\cos(2\eta))(3+\cos(2\chi))\tilde{\kappa}_{\mathrm{tr}}$	6.8E-9

Supplementary References

[1] Michelson, A. A. On the relative motion of the earth and the luminiferous ether," *Am. J. Sci.* **22,** 120–129 (1881).

[2] Michelson, A. A. & Morley, E. W. On the relative motion of the earth and the luminiferous ether, *Am. J. Sci.* **34,** 333–345 (1887).

[3] Morley, E. W. & Miller, D. C. Report of an experiment to detect the fitzgerald-lorentz effect, *Philosophical Magazine* **9,** 680–685 (1905).

[4] Tomaschek, R. Über das verhalten des lichtes außerirdischer lichtquellen, *Annalen der Physik* **378,** 105–126 (1924).

[5] Miller, D. C. Significance of the ether-drift experiments of 1925 at mount wilson, *Science* **63,** 433–443 (1926).

[6] Kennedy, R. J. A refinement of the michelson-morley experiment, *Proc. Natl. Acad. Sci. USA.* **12,** 621–629 (1926).

[7] Illingworth, K. K. A repetition of the michelson-morley experiment using kennedy's refinement, *Phys. Rev.* **30,** 692–696 (1927).

[8] Joos, G. Die jenaer wiederholung des michelsonversuchs, *Annalen der Physik* **399,** 385–407 (1930).

[9] Essen, L. A new aether-drift experiment," *Nature* **17,** 793–794 (1955).

[10] Jaseja, T. S., Javan, A., Murray, J. & Townes, C. H. Test of special relativity or of the isotropy of space by use of infrared masers," *Phys. Rev.* **133,** 1221–1225 (1964).

[11] Shamir, J. & Fox, R. A new experimental test of special relativity," *Il Nuovo Cimento B Series 10* **62,** 258–264 (1969).

[12] Brillet, A. & Hall, J. L. Improved laser test of the isotropy of space, *Phys. Rev. Lett.* **42,** 549–552 (1979).

[13] Lipa, J. A., Nissen, J. A., Wang, S., Stricker, D. A. & Avaloff, D. New limit on signals of lorentz violation in electrodynamics, *Phys. Rev. Lett.* **90,** 060403 (2003).

[14] Wolf, P. et al. Tests of lorentz invariance using a microwave resonator, *Phys. Rev. Lett.* **90,** 060402 (2003).

[15] Müller, H., Herrmann, S., Braxmaier, C., Schiller, S. & Peters, A. Modern michelson-morley experiment using cryogenic optical resonators, *Phys. Rev. Lett.* **91,** 020401 (2003).

[16] Wolf, P. et al. Improved test of lorentz invariance in electrodynamics, *Phys. Rev. D* **70,** 051902 (2004).

[17] Antonini, P., Okhapkin, M., Göklü, E. & Schiller, S. Test of constancy of speed of light with rotating cryogenic optical resonators, *Phys. Rev. A* **71,** 050101 (2005).

[18] Herrmann, S., Senger, A., Kovalchuk, E., Müller, H. & A. Peters Test of the isotropy of the speed of light using a continuously rotating optical resonator, *Phys. Rev. Lett.* **95,** 150401 (2005).

[19] Stanwix, P. L., Tobar, M. E., Wolf, P., Locke, C. R. & Ivanov, E.N. Improved test of lorentz invariance in electrodynamics using rotating cryogenic sapphire oscillators, *Phys. Rev. D* **74,** 081101 (2006).

[20] Eisele, C., Nevsky, A. Y. & Schiller, S. Laboratory test of the isotropy of light propagation at the 10^{-17} level, *Phys. Rev. Lett.* **103,** 090401 (2009).

[21] Herrmann, S. et al. Rotating optical cavity experiment testing lorentz invariance at the 10^{-17} level, *Phys. Rev. D* **80,** 105011 (2009).
